# Efficacy and safety of the single-capsule combination of fluticasone/formoterol in patients with persistent asthma: a non-inferiority trial*

**DOI:** 10.1590/S1806-37132014000600003

**Published:** 2014

**Authors:** Marti Antilla, Fábio Castro, Álvaro Cruz, Adalberto Rubin, Nelson Rosário, Rafael Stelmach

**Affiliations:** Medical Consulting Center for Clinical Research, Sorocaba, Brazil. Consultoria Médica em Pesquisa Clínica - CMPC, Medical Consulting Center for Clinical Research - Sorocaba, Brazil; Medical Consulting Center for Clinical Research, University of São Paulo, São Paulo, Brazil, University of São Paulo School of Medicine, São Paulo, Brazil; Federal University of Bahia, Salvador, Brazil. Federal University of Bahia, Salvador, Brazil; Federal University of Health Sciences of Porto Alegre, Porto Alegre, Brazil. Federal University of Health Sciences of Porto Alegre, Porto Alegre, Brazil; Federal University of Paraná, Curitiba, Brazil. Federal University of Paraná, Curitiba, Brazil; University of São Paulo, School of Medicine, São Paulo, Brazil. University of São Paulo School of Medicine, São Paulo, Brazil

**Keywords:** Asthma, Steroids, Bronchodilator agents, Administration, inhalation

## Abstract

**OBJECTIVE:**

Fluticasone and formoterol are effective in the treatment of asthma. When a corticosteroid alone fails to control asthma, combination therapy is the treatment of choice. The objective of this study was to compare the efficacy and safety of formulations containing budesonide/formoterol (BUD/FOR), fluticasone alone (FLU), and the single-capsule combination of fluticasone/formoterol (FLU/FOR) on lung function in patients with mild-to-moderate persistent asthma.

**METHODS:**

This was a randomized, multicenter, open phase III trial conducted in Brazil. The primary efficacy analysis was the assessment of non-inferiority between FLU/FOR and BUD/FOR combinations regarding FEV_1_ (in L) at the final visit. The secondary analyses were PEF, level of asthma control, serum cortisol levels, frequency of adverse events, adherence to treatment, and appropriate inhaler use.

**RESULTS:**

We randomized 243 patients to three groups: FLU/FOR (n = 79), BUD/FOR (n = 83), and FLU (n = 81). In terms of the mean FEV_1_ after 12 weeks of treatment, the difference between the FLU/FOR and BUD/FOR groups was 0.22 L (95% CI: −0.06 to 0.49), whereas the difference between the FLU/FOR and FLU groups was 0.26 L (95% CI: −0.002 to 0.52). Non-inferiority was demonstrated by the difference between the lower limits of the two 95% CIs (−0.06 vs. −0.002). The level of asthma control and PEF were significantly greater in the FLU/FOR and BUD/FOR groups than in the FLU group. There were no significant differences among the groups regarding patient adherence, patient inhaler use, or safety profile of the formulations.

**CONCLUSIONS:**

The single-capsule combination of FLU/FOR showed non-inferiority to the BUD/FOR and FLU formulations regarding efficacy and safety, making it a new treatment option for persistent asthma.

## Abstract (pt)

**OBJETIVO:**

A fluticasona e o formoterol são efetivos no tratamento da asma. A terapia combinada é o tratamento de escolha quando o corticosteroide isolado não controla a asma. O objetivo deste estudo foi comparar a eficácia e segurança de formulações contendo budesonida/formoterol (BUD/FOR), fluticasona (FLU) e fluticasona/formoterol (FLU/FOR) em cápsula única sobre a função pulmonar em pacientes com asma persistente leve e moderada.

**MÉTODOS:**

Estudo de fase III multicêntrico brasileiro, aleatorizado e aberto. A análise primária de eficácia foi a avaliação de não inferioridade da combinação FLU/FOR perante a combinação BUD/FOR em relação ao VEF_1_ (em L) na visita final. As análises secundárias foram PFE, nível de controle da asma, nível de cortisol sérico, frequência de eventos adversos, aderência ao tratamento e uso adequado do inalador.

**RESULTADOS:**

Foram randomizados 243 pacientes nos grupos FLU/FOR (n = 79), BUD/FOR (n = 83) e FLU (n = 81). Após 12 semanas de tratamento, a média da diferença do VEF_1_ foi de 0,22 L (IC95%: −0,06 a 0,49) entre os grupos FLU/FOR e BUD/FOR e de 0,26 L (IC95%: −0,002 a 0,52) entre os grupos FLU/FOR e FLU. A não inferioridade ficou demonstrada pela diferença de limite inferior do IC95% (−0,06 vs. −0,002). O nível de controle da asma e o PFE foram significativamente maiores nos grupos FLU/FOR e BUD/FOR em comparação com o grupo FLU. Não houve diferenças significativas em relação a adesão, uso do inalador e perfil de segurança entre os grupos.

**CONCLUSÕES:**

A combinação FLU/FOR em cápsula única apresentou eficácia e segurança não inferior às formulações BUD/FOR e FLU e representa uma nova opção de tratamento para asma persistente.

## Introduction

Asthma is currently one of the most common chronic diseases in the general population.^(^ 1 ^)^ It is estimated that there are approximately 300 million asthma patients worldwide, 20 million of whom are in Brazil.^(^ 2 ^)^ The central pathophysiological feature of asthma is airway inflammation, which is present in patients with mild asthma and even in asymptomatic patients.^(^ 3 ^)^ The primary focus of treatment is on reducing inflammation. 

The primary objective of asthma management is to achieve and maintain disease control.^(^ 4 ^)^ The drugs and doses used for maintenance therapy should be changed according to the level of asthma control.^(^ 1 ^)^ The ideal treatment for asthma patients is one that controls and stabilizes the disease with the lowest possible dose of medication. Inhaled corticosteroids (ICs) are the most effective anti-inflammatory drugs for the treatment of patients with weekly, daily, or continuous symptoms.^(^ 5 ^)^

The ICs that are currently available (beclomethasone, budesonide, ciclesonide, fluticasone, and mometasone) differ in terms of their potency and bioavailability; however, studies have shown that the differences have no clinical relevance.^(^ 6 ^)^ Formoterol and salmeterol are long-acting β_2_ agonists (LABAs) and should not be used as monotherapy, because they have no effect on asthmatic inflammation. They are much more effective when administered in combination with an IC.^(^ 1 ^)^

When an average dose of an IC alone fails to control asthma, combination therapy is the treatment of choice. The addition of LABAs to the daily IC dose reduces daytime and nighttime symptoms, improves lung function, reduces the need for rescue medication, and reduces the number of exacerbations,^(^ 3 ^,^ 7 ^)^ as well as promoting clinical control in a larger number of patients, doing so more quickly and with lower IC doses.^(^ 8 ^)^

The greater efficacy of combination therapy led to the development of inhalers that release fixed doses of ICs and LABAs simultaneously. Treatment with ICs and LABAs administered together is as effective as is treatment with ICs and LABAs administered separately.^(^ 9 ^)^ The use of a single inhaler containing fixed drug combinations is more convenient for patients and can therefore increase treatment adherence^(^ 10 ^)^ and guarantee that the LABA is always administered together with the IC. 

In addition to the drug itself, adherence to treatment, the type of inhaler, and the technique used in order to administer the drug are equally important for successful treatment. Poor adherence to treatment is common in asthma patients, treatment adherence rates ranging from 16% to 50%.^(^ 11 ^)^ The correct use of inhalers allows selective treatment of the lungs and reduces systemic adverse effects.^(^ 12 ^,^ 13 ^)^ The effectiveness of inhaled medications depends not only on the formulation and type of inhaler used but also on the ability of patients to use proper inhalation techniques.^(^ 14 ^)^

In a study comparing the formoterol/budesonide combination with the salmeterol/fluticasone combination, no statistically significant differences were found between the two combinations regarding the frequency of asthma exacerbations requiring emergency medical care, hospitalization, and oral corticosteroid use.^(^ 15 ^)^ These findings, however, can obscure individual differences among the components. One group of authors concluded that, when administered at half the dose of budesonide, fluticasone had a greater effect on lung function at all doses used, in all age groups studied, and for all types of devices used.^(^ 16 ^)^ Formoterol and salmeterol have been associated with different outcomes. The use of formoterol has been associated with significantly improved lung function, reduced need for rescue medication, and a greater proportion of symptom-free days when compared with the use of salmeterol.^(^ 17 ^)^

The formoterol/fluticasone combination is currently unavailable for purchase in Brazil. The experimental product containing the formoterol/fluticasone combination might be a new treatment option for asthma. The objective of the present study was to compare the efficacy and safety of budesonide/formoterol (two separate capsules), fluticasone alone, and the new, single-capsule combination of fluticasone/formoterol, all of which were delivered by dry powder inhalers (DPIs). Secondary objectives included correct inhaler use, preferred inhaler, and adherence to treatment. 

## Methods

This was a randomized, open-label phase III study conducted at six research centers in Brazil. The study population consisted of patients who met the following inclusion criteria: being 12 years of age or older; having been diagnosed with mild-to-moderate persistent asthma,^(^ 1 ^)^ with symptoms for at least 6 months; having been clinically stable for at least 1 month; having an Asthma Control Questionnaire 7 (ACQ-7) score ≤ 3^(^ 18 ^)^; currently using an IC equivalent to up to 1,000 µg of beclomethasone dipropionate, either in isolation or in combination with a LABA; and presenting with a pre-bronchodilator FEV_1_ > 40% of the predicted value. The exclusion criteria were as follows: having required hospitalization for asthma or having used oral or parenteral corticosteroids in the 3 months preceding the study; having been an active smoker in the past 3 months; presenting with severe comorbidities; having participated in other clinical studies in the past 12 months; being pregnant or lactating; and receiving chronic treatment with beta blockers. All participants gave written informed consent. The study was conducted in accordance with Good Clinical Practice guidelines and the Declaration of Helsinki.^(^ 19 ^)^

In order to increase group homogeneity and prior to randomization, patients were stratified by research center and baseline pre-bronchodilator FEV_1_: 40-70% of predicted vs. ≥ 70% of predicted. By means of a centralized electronic system, patients were randomly allocated (at a 1:1:1 ratio) to one of three groups (treatment arms): the BUD/FOR group, comprising patients receiving a combination of formoterol (12 µg) and budesonide (400 µg) delivered via an Aerolizer^(r)^-type DPI with two separate capsules (Foraseq^(r)^; Novartis Biociências S.A., São Paulo, Brazil); the FLU group, comprising patients receiving fluticasone (500 µg) delivered via a Diskus^(r)^-type multiple-dose DPI (Flixotide^(r)^; GlaxoSmithKline Brazil, Rio de Janeiro, Brazil); and the FLU/FOR group, comprising patients receiving the new, single-capsule combination of 12 µg of formoterol and 250 µg of fluticasone propionate (Eurofarma, São Paulo, Brazil) delivered via a CDM-Haler^(r)^ DPI (Emphasys Industrial, Boituva, Brazil). All inhaled drug therapies were administered twice a day (12/12 h). 

Patients were evaluated at the initial visit-the screening visit (SV)-and were randomized within 15 ± 5 days after the SV-the randomization visit (RV)-being re-evaluated at visit 1 (V1), visit 2 (V2), and the final visit (FV), 4 weeks apart. Between the SV and the RV, patients continued to use their medications, underwent measurement of cortisol levels, received a diary for PEF measurements, and, when appropriate, underwent a pregnancy test. The follow-up period was at least 12 weeks. The primary outcome measure of the present study was FEV_1_ (in L) at the FV. Secondary outcome measures included serial PEF measurements, the level of asthma control, correct inhaler use, the frequency of adverse events, and the variation in serum cortisol levels throughout the study period. At all visits after the RV, adherence to the study medication was assessed by counting the capsules/doses used. 

Spirometry was performed at all visits, in accordance with asthma management strategies.^(^ 2 ^,^ 20 ^)^ Over the course of treatment, PEF was measured in the morning and in the evening, the best of 3 measurements being recorded by patients in their diaries and subsequently evaluated by the principal investigator.^(^ 21 ^)^

Asthma control was assessed by ACQ-7 scores^(^ 18 ^)^ at the SV, at V1, at V2, and at the FV. ACQ-7 consists of seven questions regarding asthma symptom control, rescue medication use, and FEV_1_%. A score ≤ 1.57 (total score, 6 points) indicates controlled asthma. Over the course of the study, clinical assessment of asthma control was performed by the principal investigator in accordance with the Global Initiative for Asthma criteria (i.e., uncontrolled, partially controlled, and controlled asthma).^(^ 1 ^)^

Between the RV and the FV, correct inhaler use was scored on an eight-point scale. During V1 and the FV, patients answered categorical questions (yes/no questions) and one continuous variable question (graded from zero to 10) regarding the acceptability of the three inhalers and patient preference for a particular device. Serum cortisol levels were determined at the beginning and end of the study. Adverse events were recorded by the principal investigator at V1, at V2, and at the FV. 

In order to calculate the sample size, we considered that the primary outcome measure mean FEV_1_ (in L) after 12 weeks of treatment would be at least equal in all three groups in the present study. We considered a non-inferiority margin of 7.5%, which was assessed in the primary efficacy analysis comparing the FLU/FOR and BUD/FOR groups in terms of the mean FEV_1_ after 12 weeks of treatment. On the basis of literature data, we considered a mean FEV_1_ of 2.81 L, with an estimated standard deviation of 0.43 L. Considering a one-tailed type I error of 2.5% and a statistical power of 80% for detecting a maximum difference of 7.5% among the groups (non-inferiority limit), we estimated that 67 patients should be included in each treatment group. Assuming a 15% loss to follow-up, we calculated that 243 patients (81 patients per group) were required. 

Continuous variables with normal distribution were analyzed by mean and standard deviation, whereas those with non-normal distribution were analyzed by median and interquartile range. Categorical variables were described by relative frequencies. The Kolmogorov-Smirnov test was used in order to evaluate the pattern of distribution of the study variables. Continuous variables with normal distribution were compared by t-tests or ANOVA. Nonparametric tests were used for variables with non-normal distribution. The Mann-Whitney test was used for comparisons between two groups, whereas the Kruskal-Wallis test was used for comparisons among the three groups. 

## Results

A total of 273 patients were included in the present study, and 243 were randomized: 79 to the FLU/FOR group; 83 to the BUD/FOR group; and 81 to the FLU group. Of the 242 eligible patients who received treatment-1 was excluded after randomization because of the use of beta blockers-all were included in the safety population, and 235 were included in the intent-to-treat (ITT) population for having at least one evaluation of any of the study outcomes. The demographic and clinical characteristics of the patients in the ITT population are shown in Table 1. 

**Table 1 -:** Demographic and clinical characteristics of the intent-to-treat population of patients, distributed by treatment group (n = 235).a

Characteristic	Groups		
	Fluticasone/formoterol	Budesonide/formoterol	Fluticasone
	(n = 76)	(n = 82)	(n = 77)
Age, years^b^	36.72 ± 17.72	40.82 ± 16.88	39.08 ± 15.86
Gender			
Female	49 (64.5)	55 (67.1)	61 (79.2)
Male	27 (35.5)	27 (32.9)	16 (20.8)
Race			
White	47 (61.8)	52 (63.4)	43 (55.8)
Black	16 (21.1)	11 (13.4)	15 (19.5)
Mulatto	13 (17.1)	19 (23.2)	19 (24.7)
BMI,^b^ kg/m^2^	27.00 ± 5.63	27.37 ± 5.65	27.84 ± 4.57
Smoking status^c^			
Former smoker	12 (15.8)	11 (13.4)	9 (11.7)
Never smoker	64 (84.2)	71 (86.6)	68 (88.3)
Level of asthma control			
Controlled	43 (59.7)	38 (62.3)	44 (71.0)
Uncontrolled	1 (1.4)	2 (3.3)	2 (3.2)
Partially controlled	28 (38.9)	21 (34.4)	16 (25.8)
FEV_1_, L^b^	2.53 ± 0.78	2.53 ± 0.89	2.35 ± 0.59
FEV_1_, % of predicted^b^	82.07 ± 18.06	81.44 ± 17.24	81.44 ± 17.25

BMI: body mass index.

a Values expressed as n (%), except where otherwise indicated.

b Values expressed as mean ± SD.

c Had been a smoker up until three months prior to study entry.

**Figure 1 -:**
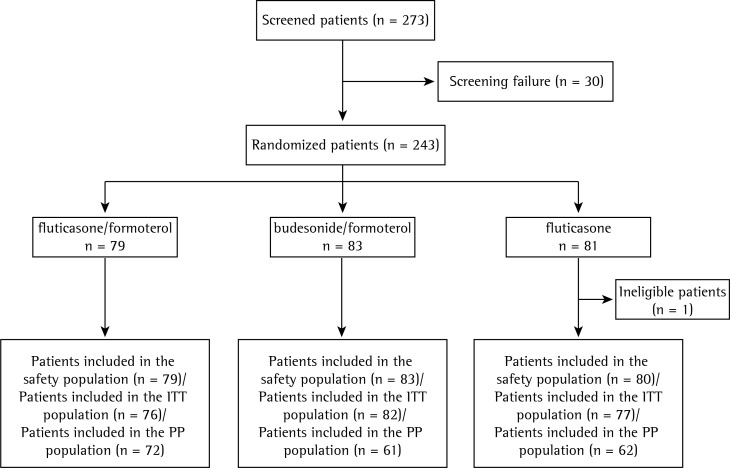
Flowchart of patients in the per-protocol (PP), intent-to-treat (ITT), and safety populations.

The per-protocol (PP) population consisted of 195 patients for whom there were FEV_1_ data at the end of the study. As can be seen in Figure 1, 7 patients were not included in the ITT population (because they missed all visits after the SV), and 47 patients were not included in the PP population (because they had no evaluation of the primary outcome, because they were excluded for failing to meet the study criteria, or because of other significant deviations). 

**Figure 2 -:**
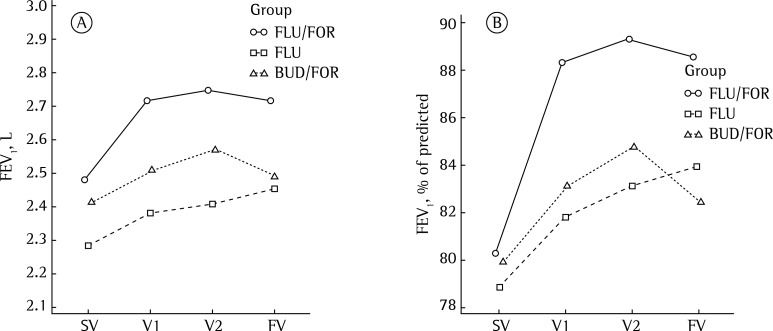
Mean FEV1 values in L (in A) and in % of predicted (in B) in the fluticasone/formoterol (FLU/ FOR), fluticasone (FLU), and budesonide/formoterol (BUD/FOR) groups (intent-to-treat [ITT] population) at each visit. SV: screening visit; V1: visit 1; V2: visit 2; and FV: final visit.

Regarding the primary outcome measure FEV_1_ after 12 weeks of treatment, the FLU/FOR, BUD/FOR, and FLU groups were similar. In the FLU/FOR, BUD/FOR, and FLU groups (ITT population), mean FEV_1_ values were 2.53 ± 0.77 L, 2.50 ± 0.87 L, and 2.41 ± 0.62 L, respectively; at the FV, mean FEV_1_ values were 2.72 ± 0.81 L, 2.50 ± 0.82 L, and 2.45 ± 0.73 L, respectively. Figure 2 shows the descriptive statistics for FEV_1_ (in L and in % of predicted) throughout the study period in all three treatment groups. Over the course of treatment, there were significant differences among assessments (p < 0.001) but no significant differences among the groups. In terms of the mean FEV_1_ after 12 weeks of treatment (PP population), the difference between the FLU/FOR and BUD/FOR groups was 0.22 L (95% CI: −0.06 to 0.48), whereas the difference between the FLU/FOR and FLU groups was 0.26 L (95% CI: −0.002 to 0.520). The primary efficacy analysis comparing the new, single-capsule combination of fluticasone/formoterol and the budesonide/formoterol combination showed non-inferiority, given that the lower limit of the 95% CI for the difference between the two combinations in terms of the mean FEV_1_ at the FV (−0.06) was greater than the pre-established non-inferiority margin (7.5% of the FEV_1_ obtained with the use of the budesonide/formoterol combination, i.e., −0.18). The secondary efficacy analysis comparing the single-capsule combination of fluticasone/formoterol and fluticasone alone also showed non-inferiority. 

Our analysis of the PEF in the morning and in the evening showed no significant differences among the groups in relation to baseline values. However, when we analyzed the combination of morning and evening PEF values throughout the study, we found significant differences among the groups (p = 0.02). We also found that the visits had an effect on PEF (p < 0.001) and that there were interactions between visits and groups (p = 0.01). In the FLU/FOR, BUD/FOR, and FLU groups, combined PEF values at the end of the study (for the ITT population) were 382.1 ± 118.4 L/s, 364.9 ± 115.2 L/s, and 324.3 ± 92.1 L/s, respectively. These results suggest that there was a gradual increase in overall PEF measurements throughout the study, especially in the FLU/FOR and BUD/FOR groups. 

**Figure 3 -:**
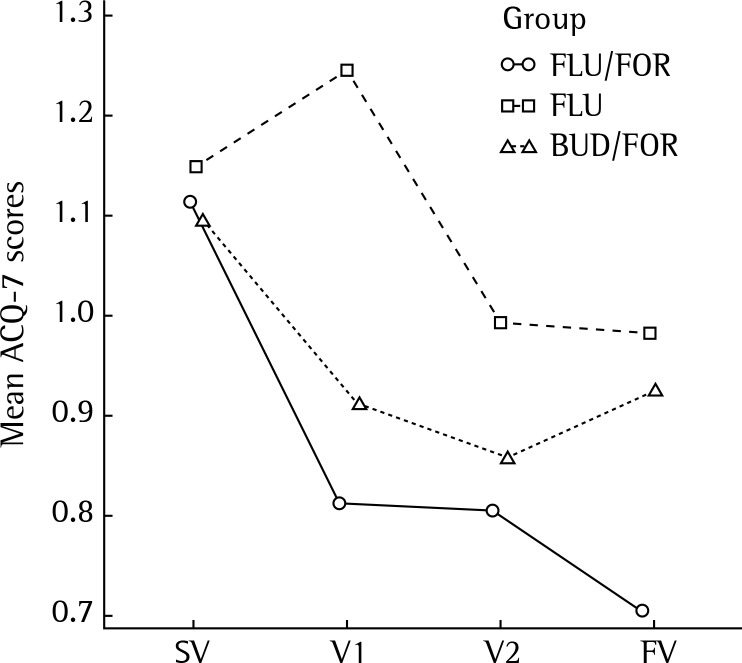
Mean Asthma Control Questionnaire 7 (ACQ-7) scores in the fluticasone/formoterol (FLU/ FOR), fluticasone (FLU), and budesonide/formoterol (BUD/FOR) groups (intent-to-treat [ITT] population) at each visit. SV: screening visit; V1: visit 1; V2: visit 2; and FV: final visit.

Figure 3 shows ACQ-7 scores per treatment group throughout the study (i.e., for the ITT population). There were statistically significant differences among treatments at V1 (p < 0.001) and at the FV (p < 0.05) but not at the RV or at V2. In the FLU/FOR, BUD/FOR, and FLU groups, mean ACQ-7 scores after 12 weeks of treatment were 0.71 ± 0.7, 0.93 ± 0.07, and 0.98 ± 0.07, respectively. Median ACQ-7 scores at the FV show that there were significant differences among the treatment groups (p < 0.05). These results of the interaction between groups and assessments suggest that although there was a gradual reduction in ACQ-7 scores throughout the study in all three groups, the reduction was more consistent in those receiving IC+LABA combinations, being more significant in the FLU/FOR group. 

Assessment of asthma control by the investigators shows that the proportions of patients with controlled asthma were similar at the beginning of the study and changed during the study. In the FLU/FOR, BUD/FOR, and FLU groups, the proportions of patients with controlled asthma at the beginning of the study were 59%, 60%, and 66%, respectively, whereas the proportions of patients with controlled asthma at the end of the study were 83%, 63%, and 68%, respectively, the difference tending to be significant (p = 0.08) in the FLU/FOR group. 

Scores > 7 (> 90% of correct answers) at all visits showed that the patients knew how to use the inhalers properly, with no differences among the groups. The decimal preference scale used showed no differences among the groups, with values above 8 points (> 80%). Table 2 shows patient adherence to treatment. In all groups, adherence to treatment varied widely between the beginning and end of the study. However, there were no differences among the groups regarding the measures of central tendency for treatment adherence. 

**Table 2 -:** Adherence to treatment in the intent-to-treat population of patients, distributed by treatment group (n = 235). Adherence

Adherence	Groups		
	Fluticasone/formoterol	Budesonide/formoterol	Fluticasone
RV to V1	(n = 75)	(n = 82)	(n = 77)
Mean ± SD	97.6 ± 12.9	94.2 ± 12.3	87.7 ± 18.0
Range	50.0-142.8	8.9-111.1	10.8-106.5
Median (IR)	98.2 (93.4-100.0)	97.0 (90.6-100.0)	93.7 (82.2-100.0)
V1 to V2	(n = 72)	(n = 78)	(n = 66)
Mean ± SD	98.1 ± 7.4	95.0 ± 7.45	95.0 ± 7.4
Range	66.6-121.1	62.5-111.1	66.3-110.0
Median (IR)	98.2 (95.8-100.0)	96.7 (90.9-100.0)	96.4 (91.8-100.0)
V2 to FV	(n = 72)	(n = 73)	(n = 62)
Mean ± SD	97.5 ± 5.9	96.8 ± 7.2	92.7 ± 11.4
Range	75.3-108.5	72.2-127.6	42.8-107.2
Median (IR)	99.4 (96.0-100.0)	98.2 (94.6-100.0)	96.35 (88.5-100.0)

RV: randomization visit; IR: interquartile range; and FV: final visit.

In the FLU/FOR, BUD/FOR, and FLU groups, the most common non-serious adverse events were asthma attacks (in 13.92%, 16.87%, and 17.50%, respectively), nasopharyngitis (in 13.92%, 21.69%, and 21.25%, respectively), and rhinitis (15.19%, 16.87%, and 25.00%, respectively). 

In the safety population (n = 242), only 2 patients in the FLU/FOR group had serious adverse events: 1 had an asthma attack related to the study treatment; and 1 had drug poisoning unrelated to the study treatment. 

Median serum cortisol levels at the end of the treatment were within the normal range, with no statistically significant differences among the FLU/FOR, BUD/FOR, and FLU groups (9.85 ± 6.46 µg/dL, 10.32 ± 5.19 µg/dL, and 9.35 ± 4.69 µg/dL, respectively). 

## Discussion

The present study compared the efficacy and safety of a single-capsule combination of fluticasone/formoterol (250 µg/12 µg, delivered via a DPI) with those of budesonide/formoterol (400 µg/12 µg, delivered via a DPI with two separate capsules) and fluticasone alone (500 µg, delivered via a multiple-dose DPI) in patients with mild to moderate asthma. The primary efficacy analysis (FEV_1_) showed that the new, single-capsule combination of fluticasone/formoterol is non-inferior to the budesonide/formoterol combination or fluticasone alone. In addition, asthma control was shown to be similar among the groups, being slightly better in the FLU/FOR group at the end of the study. The PEF at the end of the study was found to be higher in the groups of patients receiving formoterol. Patient adherence, preference, and acceptance were similar among the groups studied, as were adverse events. 

Although the non-inferiority of the fluticasone/formoterol combination was demonstrated in the present study, its superiority was not. The efficacy, safety, and tolerability of the fluticasone/formoterol combination were evaluated in a study with a design similar to ours; however, in that study, the drugs were delivered via pressurized metered dose inhalers (MDIs).^(^ 22 ^)^ The combination of fluticasone/formoterol delivered via a single pressurized MDI, the fluticasone/formoterol combination delivered via two separate MDIs, and fluticasone alone were also found to be non-inferior to one another in terms of FEV_1_ values and safety outcomes. Post hoc analyses of the two studies showed significant differences in symptoms, asthma control, and PEF between the single-capsule combination of fluticasone/formoterol delivered via a DPI and the fluticasone/formoterol combination delivered via a single pressurized MDI, especially in comparison with fluticasone alone. Studies involving a larger number of patients might show these differences more clearly. 

It has been demonstrated that fluticasone/formoterol delivered via a single MDI is non-inferior to fluticasone/salmeterol delivered via a multiple-dose inhaler.^(^ 23 ^)^ However, the response to treatment was faster in the group of patients receiving formoterol, with a possible impact on patient preference and adherence. In the present study, two groups of patients received formoterol, and it was therefore impossible to distinguish clearly between the two. Patient preference for, acceptability of, and mean/median adherence to the different devices were similar in the present study. However, as can be seen in Table 2, the lowest adherence rates at the end of the study in the FLU/FOR and BUD/FOR groups were superior to those in the group of patients receiving an IC alone. 

Two meta-analyses^(^ 24 ^,^ 25 ^)^ and one systematic review^(^ 17 ^)^ showed that the use of fluticasone is associated with better outcomes than is the use of budesonide. Despite the small number of comparative studies at the time, fluticasone was associated with better prevention of asthma exacerbations.^(^ 17 ^)^ Another difference observed is the need for higher doses of budesonide in order to achieve similar asthma control, probably because the fluticasone molecule has a higher binding affinity to the corticosteroid receptor and because of their different pharmacokinetic characteristics.^(^ 26 ^)^

The efficacy of any inhaled asthma therapy in clinical practice depends on a number of factors, including the efficacy of the drug, the amount of drug released, correct inhaler use, the inhalation technique, and adherence to treatment. Poor adherence to maintenance treatment and the lack of asthma control in nearly 50% of the studies (including research protocols) appear to be associated with the aforementioned factors. More than 100 inhaler/drug combinations were available 5 years ago. ^(^ 27 ^)^ Since then, the number of new drugs and inhalers has increased, and hydrofluoroalkane has emerged. The criteria for selecting the best inhaler vary among manufacturers, patients, and physicians.^(^ 28 ^)^ In the real world, clinical efficacy, local availability of the device, the price of the device, and patient preference/acceptance should be taken into consideration. Our decision to use a single-capsule combination of fluticasone/formoterol delivered twice daily via a DPI appears to have contributed to treatment adherence. DPIs are easier to use than are MDIs.^(^ 29 ^)^

The inhaler used in the present study in order to deliver the new, single-capsule combination of fluticasone/formoterol (i.e., the CDM-Haler^(r)^ inhaler) was tested in vitro against the Diskus^(r)^ inhaler in order to determine the PEFs generated at 4 kPa at constant volume, as well as dose uniformity and aerodynamic particle size distribution. The results showed dose uniformity and uniform aerodynamic particle size distribution at the beginning, middle, and end of 360 actuations in 10 of the inhalers tested.^(^ 30 ^)^

The strength of the present study is its design, which is consistent with other, similar, multicenter non-inferiority studies. The methodology allowed us to use ITT analysis and PP analysis to assess the study population and the primary outcome, respectively. However, the study might have been more robust if we had included only patients with severe uncontrolled asthma. The decision to exclude such patients was deliberate, given that the number of patients required in order to achieve non-inferiority would have been much larger, with a screening failure higher than the observed rate of 15%. The use of asthma control measures by the patients and investigators, as well as objective assessments of treatment adherence and inhaler use, would have allowed us to detect signs of efficacy in part of the study population, allowing inferences about the personalization of asthma treatment. 

National studies of new drugs (phase III studies) are important in order to analyze specific responses in the study population. Regulatory agencies in countries such as Japan and China do not allow the marketing of new drugs until national studies have been conducted, given that individual response to treatment varies across patient populations. In Brazil, there have been few multicenter studies in the respiratory field involving a large number of patients. The results of the present study show the importance of such studies, especially when they are aimed at providing drugs whose formulation might improve patient acceptance, preference, and adherence. 

In conclusion, the present study demonstrated that the new, single-capsule combination of fluticasone/formoterol is non-inferior to reference drugs budesonide/formoterol and fluticasone. In addition, the fluticasone/formoterol combination showed comparable efficacy and safety, being able to control persistent asthma, as determined by objective assessment of the use of and adherence to inhaled asthma therapy. The use of this IC+LABA combination as sole maintenance therapy represents an effective treatment option for patients with persistent asthma, especially those with an inadequate response to ICs in isolation or other IC+LABA combinations. 
